# Predictors of end-of-life care stress, calling, and resilience on end-of-life care performance: a descriptive correlational study

**DOI:** 10.1186/s12904-022-00961-0

**Published:** 2022-05-17

**Authors:** Ji-Young Kim, Eun-Hi Choi

**Affiliations:** 1 Deajeon Eulji Medical Center, Daejeon, Republic of Korea; 2 Department of Nursing, Eulji University, Gyeonggi, Republic of Korea

**Keywords:** End-of-life care, Palliative care nursing, Stress, Calling, Resilience

## Abstract

**Background:**

Prolonging the end-of-life process means that the duration of health care work increases and the management of death is delegated to health care providers by patients’ families. Thus, it is important to explore measures to enhance the quality of end-of-life care by identifying the predictors thereof. End-of-life care should be people-centred, relieving serious health-related suffering, be it physical, psychological, social, or spiritual. Nurses who provide end-of-life care usually spend the most time with dying patients, administering care to help patients who wish to die with dignity; therefore, end-of-life nursing care is highly significant.

**Methods:**

This study was conducted on nurses of 500-bed or larger university hospitals in city D and province C in South Korea from 20 August to 10 September 2020 using a structured questionnaire. A total of 213 nurses with a minimum clinical career of one year and at least one EOLC experience participated. The final analysis sample consisted of 206 nurses. Descriptive analysis, Pearson’s correlation coefficients, ANOVA, t-test, and multiple-regression analysis were used to analyse the data.

**Results:**

This study found that end-of-life care performance was significantly positively correlated with end-of-life care stress [*r* = .253, *p* < .001], sense of calling [*r* = .424, *p* < .001], and resilience [*r* = .397, *p* < .001]. End-of-life care stress [β = .185, *p* = .003], sense of calling [β = .259, *p* < .001], resilience [β = .252, *p* < .001], and working in a hospice ward [β = .140, *p* = .041] or intensive care unit [β = .218, *p* = .008], as opposed to the emergency department, were identified as predictors of end-of-life care performance. These factors explained 28.3% of the variance in the end-of-life care performance in this study.

**Conclusions:**

Boosting the sense of calling and resilience among nurses providing palliative care can improve overall end-of-life care performances. Subsequent studies should develop and evaluate interventions and programs that could improve these factors to ensure a positive change in health care and enhance the quality of end-of-life care in hospitals.

## Background

Recent advances in medical technology and life-sustaining medical equipment have contributed substantially to extending lives; however, in some aspects, these advances only postpone the time of death of irrecoverable patients instead of extending quality life years, which has led to an increase in the number of people spending their final years of life in health care facilities [1, 2]. Prolonging the dying process means that the duration of health care work is extended and that patients’ families delegate the management of death to health care providers.

For Korean people, dying with dignity refers to dying at peace by preparing for death through interactions with family and health care providers to avoid a meaningless extension of life [3, 4]. End-of-life care [EOLC] should be people-centred, relieving serious health-related suffering, be it physical, psychological, social, or spiritual [5]. Thus, health care providers play a key role in helping patients who spend their final years of life at a health care facility and their families prepare for a dignified death [6]. Nurses who provide EOLC usually spend the most time with dying patients, administering EOLC to help patients who wish to die with dignity; therefore, it is highly significant.

The traditional Korean society discourages mentioning the impending death of an end-of-life patient as it is taboo, but people often wish to spend their last days with their family [7]. Additionally, dying patients might experience a sense of regret and an unending desire to live while they wait for their final moment [8]. Their family members might also feel a sense of regret and fatigue as they watch and take care of the dying patients who suffer from anxiety and pain [9]. When healthcare workers care for dying patients, medical decisions are often made by family members only, as opposed to the patients [10].

However, while providing care for dying patients and their families, nurses experience EOLC stress which encompasses physiological, psychological, and social burdens [2, 11]. Nurses experience high levels of stress when providing EOLC, unlike when providing general care, due to factors such as high workloads, conflicts regarding the limitations of medicine, negative attitudes toward death, and lack of EOLC experience, as well as physical fatigue and psychological pain, such as helplessness for not being able to do anything to save the patient, unrewarded dedication, and despair when a patient dies [6, 12]. Thus, nurses’ EOLC stress must be reduced to enhance the quality of care they can provide.

Other variables that may influence EOLC or related stress are nurses’ resilience and the senses of calling. Resilience refers to the ability to respond flexibly and adjust successfully to situational demands or stressful environments and grow through and overcome challenges based on appropriate self-regulation [13]. This quality offsets the impact of stress and facilitates a certain level of functioning and adjustment despite various difficulties and excessive workloads [14]. Previous studies have reported that psychological emptiness and diminishing inner balance influence nurses’ resilience [15] and that while nurses with low resilience have difficulty enduring stress and its negative effects, nurses with high resilience are at a lower risk of burnout during work [16] and can cope with stress more effectively, which increases their work efficiency and nursing expertise [17]. Hence, it is necessary to investigate whether resilience influences EOLC-related stress.

Calling refers to the sense of fulfilment and purpose an individual feels about their work that drives their work dedication; it is closely linked to the nursing profession, which deals with human lives [18]. The nursing profession is characterised by pride, and people with a sense of calling are satisfied with their work and have the strength to endure and overcome challenges [19]. Studies have found that people who consider their job as a calling show high commitment and satisfaction with their jobs, so nurses with a calling are likely to demonstrate active attitudes in their jobs [20]. From this perspective, it is important to examine how nurses’ senses of calling affect their EOLC.

Thus, there is a need for studies that examine the level of EOLC stress and the correlations between EOLC stress, calling, and resilience in nurses who provide EOLC to determine whether these factors can enhance it. This study is significant as it presents foundational data for developing interventions for stress, calling, and resilience to promote quality EOLC.

This study aims to investigate the effects of EOLC stress, calling, and resilience on EOLC performance in nurses, with the following specific objectives: First, we examine the levels of EOLC stress, calling, resilience, and EOLC performance among the participants. Second, the levels of EOLC performance according to participants’ characteristics are examined. Third, we present the correlations between EOLC stress, calling, resilience, and EOLC performance. Lastly, the predictors of EOLC performance are identified.

## Methods

### Design

This study is a descriptive correlational investigation aimed at examining the level of EOLC stress, calling, and resilience, and their impact on EOLC performance in nurses.

### Sample and power analysis

Nurses with at least one EOLC experience and one year in a clinical career who worked in a 500-bed or larger university hospital in city D and province C were enrolled in this study.

Using the G*power 3.1.9.4 program, the minimum sample size required for regression analysis with a medium effect size of 0.15, as proposed by Cohen [21], a significance level of 0.05, power of 0.95, and 14 predicting variables was 194. Considering a 10% dropout rate, the questionnaire was distributed to 213 participants, and after excluding seven questionnaires due to careless responses, a total of 206 questionnaires were included in the final analyses. Permission for data collection was obtained from the nursing departments of the included university hospitals in city D and province C, and the questionnaire was administered only to those who consented to participate. The self-reported questionnaire took approximately 10 min to complete, and data were collected from 20 August to 10 September 2020.

### Ethical approval and consent to participate

This study was approved by the Institutional Review Board of Eulji University (EUN20-033) before data collection. The participants were provided with an information sheet and consent form specifying the anonymous nature of the survey and their freedom to withdraw from the study at any time. In this study, informed consent was obtained from all subjects to participate in the study and all methods were carried out in accordance with relevant guidelines and regulations stipulated in Declaration of Helsinki. Only those who signed the consent form were enrolled in the study. The participants placed their completed questionnaire in a sealed envelope to submit to the researcher, and those who participated in the questionnaire were given a small gift.

### Instruments

#### End-of-life care (EOLC) stress

EOLC stress refers to the stress experienced by nurses from physiological, psychological, and social burdens while providing care for patients at the end of their lives and their families [11]. In this study, we used the EOLC Stress Scale developed by Lee [22]. This tool contains 42 items for seven subscales, with 10 items for negative attitude toward death of patient/caregiver, eight items for difficulty allotting enough time for EOLC, six items for burden of EOLC, five items for excessive workload, six items for interpersonal conflicts with the dying patient, three items for inadequate expert knowledge and skills, and four items for conflicts regarding the limitations of medicine. Each item is rated on a 5-point Likert scale ranging from strongly disagree (1) to strongly agree (5), with a higher score indicating greater EOLC stress. The Cronbach’s α was 0.93 in the study by Lee et al. and in this study.

#### Resilience

Resilience refers to an individual’s psychosocial ability and quality that enables successful adjustment to imminent adversity, recovery to a normal state, and further growth [13]. In this study, we used the Korean version of the Connor-Davidson Resilience Scale (KCD-RISC) adapted and validated by Baek [23] based on the original Connor-Davidson Resilience Scale (CD-RISC) developed by Connor and Davidson [24]. This 25-item tool contains nine items for tenacity, eight items for persistence, four items for optimism, two items for support, and two items for spirituality. Each item is rated on a 5-point Likert scale ranging from strongly disagree (0) to strongly agree (4), with a higher score indicating greater resilience. The Cronbach’s α was 0.89 in the study by Baek et al., and 0.93 in this study.

#### Calling

A calling is how an individual perceives their vocation, realises their roles in their work, seeks meaning and purpose through work, and ultimately seeks to exert a positive influence for the good of the public [18]. We used the Korean version of the Calling and Vocation Questionnaire (CVQ) developed by Dik et al. [18] and adapted to Korean and further validated by Shim and Yoo [25]. This tool contains 12 items, with four items each for transcendent summons, deriving or expressing meaning or purpose through work, and prosocial orientation in work. Each item is rated on a 4-point Likert scale from not at all true of me (1) to absolutely true of me (4), with a higher score indicating greater calling. The Cronbach’s α was 0.91 in the study by Yoo and Shim and this study.

#### End-of-life care (EOLC)

EOLC refers to comprehensive care that addresses the physical, psychological, and spiritual needs of patients at the end of their lives and their families to help them maintain a high quality of life and die peacefully with dignity [5]. In this study, we used the End-of-Life Care tool developed by Park and Choi [26]. The tool has to check the nursing care that the nurse provides to the patient. This tool contains 22 items in three dimensions, with eight items for the physical dimension, eight items for the psychological dimension, and six items for the spiritual dimension. Physical dimensions included questions about partial baths, oral nursing, position changes, dietary intake, skin care, and support for use, etc. Psychological dimensions included questions such as dialogue, courtesy, together when desired, and promoting value, etc. Spiritual dimensions included faith or value respect, talk about the meaning and purpose of life together, and read the scriptures of patient religion, etc. Each item is rated on a 4-point Likert scale from never (1) to always (2), with a higher score indicating greater EOLC performance. The Cronbach’s α was 0.93 in the study by Park et al., and 0.85 in this study.

### Data analysis

SPSS WIN 26.0 software was used for analyses. Participants’ characteristics were analysed using descriptive statistics. The levels of EOLC stress, calling, resilience, and EOLC performance are presented as means and standard deviations. The differences in EOLC performance according to participants’ characteristics were analysed using t-tests and ANOVA, followed by the Scheffé test for post-hoc comparison.

The relationships between EOLC stress, calling, resilience, and EOLC performance were analysed using Pearson’s correlation coefficients. The predictors of EOLC performance were analysed using multiple regression analysis.

## Results

### Participants’ general characteristics

Table 1 shows the characteristics of the participants. Of the 206 participants, 6.3% were men, and 93.7% were women. The mean age was 29.94 years, with 17% aged 25 years or younger, 67% between 26 and 35 years, and 16% aged 36 years or older. A total of 79.1% were single, and 62.1% did not have a religion. The majority of the participants had a bachelor’s degree (75.7%).

**Table 1:** Characteristics of Participants (*n* = 206)

**Characteristics**	**Categories**	***N*(%)**	**M ± SD**
Gender	Male	13(6.3)	
	Female	193(93.7)	
Age (years)	≤ 25	35(17.0)	29.94 ± 5.35
	26 ~ 35	138(67.0)	
	≥ 36	33(16.0)	
Marital status	Married	43(20.9)	
	Single	163(79.1)	
Religion	Yes	78(37.9)	
	No	128(62.1)	
Educational level	College	24(11.7)	
	University	156(75.7)	
	Graduate school or higher	26(12.6)	
Clinical experience	< 2	35(17.0)	6.61 ± 5.25
	2 ~ < 6	81(39.3)	
	6 ~ < 10	45(21.8)	
	≥ 10	45(21.8)	
Job position	Staff nurse	185(89.8)	
	≥ Charge nurse	21(10.2)	
Work unit	Emergency room	35(17.0)	
	Intensive care unit	53(25.7)	
	General ward	105(51.0)	
	Hospice unit	13(6.3)	
Number of end-of-life care experiences	≥ 2	37(18.0)	13.91 ± 20.31
	3 ~ 10	110(53.4)	
	11 ~ 20	25(12.1)	
	> 20	34(16.5)	
Experience of end-of-life for close family member or friend	Yes	99(48.1)	
	No	107(51.9)	
Experience of hospice education	Yes	113(54.9)	
	No	93(45.1)	

The mean length of career was 6.61 years, and 89.8% were staff nurses. Work units included general ward (51.0%), intensive care unit (ICU) (25.7%), emergency department (ED) (17.0%), and hospice ward (6.3%). The number of instances where EOLC was provided in the past year was two or fewer (18.0%) or three to ten (53.4%). A total of 51.9% had never witnessed the death of a close family member or friend, and 54.9% had prior hospice/EOLC education.

### Levels of EOLC stress, calling, resilience, and EOLC performance

Table 2 shows the levels of EOLC stress, EOLC performance, calling, and resilience. The mean EOLC stress score was 3.68 out of 5. The mean calling score was 2.29 out of 4. The mean resilience score was 2.44 out of 4. The mean EOLC performance score was 2.31 out of 4.

**Table 2:** Levels of Terminal care stress, calling, resilience, terminal care performance (*n* = 206)

**Variables (Item No.)**	**Item score**	**Totat score**	**Min**	**Max**	**Range**
	**M ± SD**	**M ± SD**			
**EOLC stress (42)**	3.68 ± 0.51	154.69 ± 21.27	89	210	42 ~ 210
Negative attitude of patient and his/her family members (10)	3.80 ± 0.63	38.00 ± 6.25	17	50	10 ~ 50
Burden about bereavement care (8)	3.62 ± 0.59	28.94 ± 4.74	16	40	8 ~ 40
Difficulty of allocating time to dying patient (6)	3.29 ± 0.77	19.73 ± 4.60	8	30	6 ~ 30
Overloaded duty (5)	3.80 ± 0.66	18.98 ± 3.30	5	25	5 ~ 25
Conflict about limitations of medicine (6)	3.77 ± 0.70	22.61 ± 4.19	10	30	6 ~ 30
Human relation conflict with dying patient (3)	3.78 ± 1.14	11.33 ± 3.44	5	53	3 ~ 15
Insufficiency in professional knowledge and skill (4)	3.77 ± 0.62	15.10 ± 2.48	6	20	4 ~ 20
**Calling (12)**	2.29 ± 0.59	27.46 ± 7.13	13	48	12 ~ 48
Transcendent summons (4)	2.00 ± 0.70	8.01 ± 2.82	4	16	4 ~ 16
Purpose/meaning (4)	2.42 ± 0.69	9.68 ± 2.78	4	16	4 ~ 16
Prosocial orientation (4)	2.44 ± 0.68	9.76 ± 2.72	4	16	4 ~ 16
**Resilience (25)**	2.44 ± 0.53	60.91 ± 13.17	26	96	0 ~ 100
Hardiness (9)	2.32 ± 0.61	20.84 ± 5.49	8	35	0 ~ 36
Persistence (8)	2.50 ± 0.61	20.03 ± 4.92	8	32	0 ~ 32
Optimism (4)	2.42 ± 0.67	9.67 ± 2.66	3	16	0 ~ 16
Support (2)	3.16 ± 0.61	6.31 ± 1.22	1	8	0 ~ 8
Spiritual influence (2)	2.03 ± 0.76	4.07 ± 1.52	1	8	0 ~ 8
**EOLC performance (22)**	2.31 ± 0.35	50.85 ± 7.77	32	71	22 ~ 88
Physical area (8)	2.60 ± 0.53	20.84 ± 4.22	8	32	8 ~ 32
Psychological area (8)	2.67 ± 0.47	21.38 ± 3.80	10	32	8 ~ 32
Spiritual area (6)	1.44 ± 0.42	8.64 ± 2.51	6	18	6 ~ 24

### Differences in EOLC performance according to participants’ characteristics

Table 3 shows the differences in EOLC performance according to participant characteristics. EOLC performance significantly differed according to age (*F* = 3.039, *p* = 0.050), education level (*F* = 3.874, *p* = 0.022), work unit (*F* = 3.179, *p* = 0.025), and witnessing the death of a close family or friend (*F* = 2.284, *p* = 0.023).

**Table 3:** Difference of EOLC performance according to nurses’ characteristics (*n* = 206)

Characteristics	Categories	*N*	M ± SD	*t/F*	*p*
					**(scheffe)**
Gender	Male	13	48.46 ± 9.26	-0.972	0.349
	Female	193	51.02 ± 7.66		
Age (years)	≤ 25^b^	35	52.66 ± 6.81	3.039	**0.050** **(a < b)**
	26 ~ 35^a^	138	49.93 ± 7.80		
	≥ 36^b^	33	52.82 ± 8.09		
Marital status	Married	43	52.16 ± 8.20	1.193	0.237
	Single	163	50.51 ± 7.64		
Religion	Yes	78	50.05 ± 7.87	-1.153	0.251
	No	128	51.34 ± 7.69		
Educational level	College^a^	24	51.79 ± 10.03	3.874	**0.022** **(b < c)**
	University^b^	156	50.10 ± 7.22		
	Graduate school or higher^c^	26	54.50 ± 7.76		
Clinical experience	< 2	35	51.40 ± 7.26	0.187	0.905
	2 ~ < 6	81	50.44 ± 7.40		
	6 ~ < 10	45	50.71 ± 8.97		
	≥ 10	45	51.31 ± 7.72		
Job position	staff nurse	185	50.58 ± 7.81	-1.644	0.112
	≥ charge nurse	21	53.29 ± 7.07		
Work unit	Emergency room^a^	35	48.37 ± 8.12	3.179	**0.025** **(a < d)**
	Intensive care unit^b^	53	52.81 ± 6.74		
	General ward^c^	105	50.33 ± 7.74		
	Hospice unit^d^	13	53.77 ± 9.01		
Number of end-of-life care experiences	≥ 2	37	51.89 ± 7.11	1.834	0.142
	3 ~ 10	110	49.84 ± 7.59		
	11 ~ 20	25	53.48 ± 9.08		
	> 20	34	51.09 ± 7.70		
Experience of end-of-life of family member or friend	Yes	99	52.13 ± 8.08	2.284	**0.023**
	No	107	49.67 ± 7.31		
Experience of hospice education	Yes	113	51.08 ± 7.58	0.455	0.649
	No	93	50.58 ± 8.03		

Values with superscript letters a,b, c and d are signifcantly diferent acorss rows (*p* < .05)

### Correlations between EOLC stress, calling, resilience, and EOLC performance

Table 4 shows the correlations between EOLC stress, calling, resilience, and EOLC performance. EOLC performance was significantly correlated with calling (*r* = 0.424, *p* < 0.001), resilience (*r* = 0.397, *p* < 0.001), and EOLC stress (*r* = 0.253, *p* < 0.001). This suggests that EOLC performance increases with increasing EOLC stress, calling, and resilience.

**Table 4:** Correlation between EOLC care stress, calling, resilience and EOLC performance (*n* = 206)

Variables	Physical	Psychological	Spiritual	EOLC performance
	**r (p)**	**r (p)**	**r (p)**	**r (p)**
EOLC stress				0.253(< .001)
Negative attitude of patient and his/her family members	0.112 (.109)	0.296(< .001)	0.160(.021)	
Burden about bereavement care	0.103 (.142)	0.272(< .001)	0.173(.013)	
Difficulty of allocating time to dying patient	0.126 (.072)	0.226(.001)	0.165(.018)	
Overloaded duty	-0.035 (.618)	0.132(.058)	0.125(.073)	
Conflict about limitations of medicine	0.143 (.040)	0.261(< .001)	0.090(.196)	
Human relation conflict with dying patient	0.038 (.592)	0.026(.712)	-0.006(.934)	
Insufficiency in professional knowledge and skill	0.096 (.170)	0.033(.634)	-0.009(.896)	
Calling				0.424(< .001)
Transcendent summons	0.197 (.005)	0.224(.001)	0.250(< .001)	
Purpose/meaning	0.332(< .001)	0.335(< .001)	0.240(.001)	
Prosocial orientation	0.305(< .001)	0.296(< .001)	0.193(.005)	
Resilience				0.397(< .001)
Hardiness	0.241(< .001)	0.289(< .001)	0.191(.006)	
Persistence	0.273(< .001)	0.387(< .001)	0.220(.001)	
Optimism	0.228(.001)	0.255(< .001)	0.164(.019)	
Support	0.213(.002)	0.220(.002)	-0.041(.560)	
Spiritual influence	0.180(.010)	0.119(.088)	0.186(.007)	

### Predictors of EOLC stress, calling, and resilience on EOLC performance

Table 5 shows the results of the multiple regression analysis to identify the predictors of EOLC performance. Age, work unit, and experience of loved one’s death, which significantly differed from participants’ characteristics, were entered as independent variables, and work unit and experience of loved one’s death were dummy-coded for the analysis. The regression equation was significant, indicating that EOLC stress, calling, and resilience significantly predicted EOLC performance (*F* = 11.113, *p* < 0.001), and the independent variables explained 28.3% of the variance in EOLC performance. The variance inflation factor (VIF) was below 10 at 1.042–2.117 and the Durbin-Watson index was 2.117, confirming the absence of multicollinearity and autocorrelation, respectively.

**Table 5:** Factors influencing EOLC performance (*n* = 206)

	**B**	**SE**	**β**	***t***	***p***	***VIF***
(Constant)	21.745	4.388		4.956	< .001	
Age	0.043	0.089	-0.030	-0.484	.629	1.081
EOLC stress	0.067	0.023	0.185	2.988	.003	1.093
Calling	0.282	0.079	0.259	3.569	< .001	1.504
Resilience	0.149	0.041	0.252	3.601	< .001	1.402
Work unit (Hospice unit)^a^	4.454	2.162	0.140	2.060	.041	1.316
Work unit (Intensive care unit)^a^	3.868	1.446	0.218	2.675	.008	1.903
Work unit (General ward)^a^	1.981	1.334	0.128	1.485	.139	2.117
Experience of end-of-life (Yes)^a^	1.817	0.936	0.117	1.941	.054	1.042
*R*^2^ = 0.311, Adjusted *R*^2^ = 0.283, *F* = 11.113, *p* < .001						
Durbin-Watson = 2.117						

^a^ Dummy variables = Work unit (Emergency room: 0; Hospice unit, Intensive care unit, and General ward: 1); Experience of end-of-life (No:0, Yes:1)

Calling had the greatest effect on EOLC performance (β = 0.282, *p* = 0.001), followed by EOLC stress (β = 0.185, *p* = 0.003) and resilience (β = 0.149, *p* < 0.001), while the EOLC performance was higher among nurses who worked in a hospice ward (β = 0.140, *p* = 0.041] and those who worked in the ICU (β = 0.218, *p* = 0.008) compared to those who worked in the ED.

## Discussion

This study aimed to investigate the effects of EOLC stress, calling, and resilience on EOLC performance to present foundational data for exploring measures to improve nurses’ EOLC care. The results are discussed below.

The mean EOLC performance score was 2.31 out of 4, with the highest score being for the psychological dimension, followed by the physical and spiritual dimensions. This supports previous results stating that the quality of spiritual care among nurses is poor [27]. In an end-of-life situation, spiritual care is rarely provided, and most nurses avoid spiritual care due to inadequate preparation and training [28], with most nurses equating spiritual care with religion. Patients receiving EOLC and their families show spiritual care needs, therefore, it is important to increase the spiritual care provided by nurses to them [29]. These results highlight the importance of improving nurses’ competencies in the spiritual domain, as similarly reported in previous studies [30, 31]. In addition to physical care, such as pain relief, religious care, such as reading the Bible and providing counselling with a spiritual leader, can enhance the quality of death for end-of-life patients [32]. Based on the nurse’s condition and circumstances, spiritual care might include three dimensions of relationship: with a supernatural being, with others, and with oneself [33]. Therefore, it is essential to include the spiritual content when developing nurse training courses or programs for end-of-life care. Simultaneously, it is crucial to improve the conditions of the hospital for spiritual care to become available. Further, tools to assess nursing needs in the spiritual domain may be developed to help create and implement nursing interventions, which in turn would enhance the quality of EOLC.

In this study, calling was the most potent predictor of EOLC performance. This suggests that having a sense of calling, which would enable nurses to feel more responsible, as opposed to feeling burdened, in response to various care needs of patients and their families in an end-of-life situation, would increase the quality of care provided by nurses. Having a calling allows nurses to accept their job and work as a part of their lives and thus help them be successful in their work [34]; hence, calling has a positive effect on nurses’ work performances [35] and is an essential attribute for nurses to improve their EOLC performance. Calling contributes to improved work meaningfulness, work engagement, career commitment, personal well-being, and satisfaction by promoting personal introspection and cognitive awareness [36]. It was reported that organizational support and job crafting are particularly important elements of a work environment that encourages nurses to experience a sense of calling [37]. Therefore, to foster nurses’ senses of calling, organisations may provide internal motivation, offer education opportunities, and acknowledge nursing as a profession such that nurses are recognised for their professional roles.

Resilience also predicted EOLC performance, where nurses’ EOLC performances increased with increasing resilience. Resilience develops when individuals have supporters who stand by their sides during difficult times [38, 39]. Support is provided through significant social relationships, such as family, friends, and colleagues [40], and resilience can be altered by organisational interventions, such as learning and training, by interacting with the environment when facing stress or crises [41]. These results shed light on the need for programs that increase organisational coherence through workshops and mentoring, to promote interactions among colleagues in consideration of the nature of nurses’ work that involves interdisciplinary collaboration and interaction within the hospital.

Next, it was found that EOLC stress predicted EOLC performance. Providing EOLC is a highly stressful event for nurses, and they experience high levels of stress due to role conflicts, psychological burdens, and time-pressing work as their expertise and clinical competencies increase. Although past findings on stress and performance are somewhat inconsistent, an experimental study reported that stress caused by challenges in task-solving actually improves performance [42]. This reflects the Yerkes-Dodson law [43], which explains that an appropriate level of stress is conducive to creative activities and enables problem-solving. Conversely, increased anxiety due to stress responses can diminish one’s performance [42]. As repetitive simulation training reduces anxiety [44], practical education programs may be developed to boost EOLC performance. Furthermore, because persistently high levels of stress induce burnout [45], stress should be managed to be maintained at a certain level during EOLC, and the factors associated with EOLC stress may be identified. As a cross-sectional study, the method adopted does not clearly show a causal relationship. It is probable that the increase in workload of EOLC could have led to the increase in EOLC stress. Moreover, during the COVID-19 pandemic, when this study was conducted, higher levels of stress, exhaustion, and depressive mood, as well as lower levels of work-related fulfilment were reported [46]. Thus, it is necessary to make efforts to relieve the stress that nurses experience even if they put a high level of effort to perform their nursing jobs to the best of their ability and fulfil their calling as healthcare professionals.

In this study, there was a significant difference in EOLC performance between nurses with only one and those with two or more EOLC experiences per year. As nurses with less EOLC experiences fear of EOLC and are less skilled in their approaches, a support system may be developed through which nurses with more EOLC experiences can share their skills with less experienced nurses. The work unit in which nurses worked also predicted EOLC performance, where nurses who worked in the hospice ward and ICU showed significantly better EOLC performance than those who worked in the ED. This matched the findings of previous studies showing that such disparity stems from the differences in the departments in terms of intensity and medical treatment [47]. This may be attributable to the fact that hospice ward nurses frequently provide EOLC and experience patient deaths, and ICU nurses provide patient care without the assistance of patients’ caregivers, as opposed to ED nurses who are required to provide emergency and acute care [48]. Staff nurses lack awareness of palliative care [49], but nurses from the ICU or hospice ward experience EOLC more often, which might have increased their awareness of palliative care. Thus, education and training programs for nurses providing EOLC may be tailored to specific work units. ICU nurses showed increased competency in palliative care with more service training in palliative care or EOLC [50]. Previous studies observed high needs for role definition and practice authority for advanced practice nurses [49] and a need for education [51]. Studies also reported that preparations for EOLC are lacking and that patients, caregivers, and health care providers may be educated about writing advance directives, symptoms at the end of life, and communication [52].

The limitation of this study is that it is restricted in generalization to nurses in one area, that is, South Korea. This is because each country has a different culture of end-of-life care. Based on these results, we suggest the following: Customised EOLC training manuals tailored to each work unit can be developed for application in clinical practice. Further exploratory studies that include other factors of EOLC may be conducted. A feasible instrument to assess care needs in the spiritual domain may be developed to enhance EOLC of nurses as this domain showed great significance in this study. Finally, support systems and education programs that help nurses develop effective coping strategies for EOLC stress, calling, and resilience may be developed, applied in stages according to job positions, and evaluated to ensure their impact.

## Conclusion

This study is a descriptive correlational investigation aimed at determining the effects of EOLC stress, calling, and resilience on EOLC performance in nurses. The ultimate objective was to present foundational data for developing intervention programs to enhance the quality of EOLC.

This study has implications for nursing practices as it is the first study to investigate the relationship between EOLC stress, calling, and resilience in nurses providing EOLC, and the findings would be useful to improve EOLC. However, this study was conducted with nurses from a university hospital, and nurses without prior EOLC experience and less than one year of clinical experience were excluded; therefore, the findings should be generalised with caution.

The following results were obtained: EOLC performance differed significantly according to age, education level, work unit, and experience of death of a loved one, and EOLC stress significantly differed according to age, career, job position, and number of EOLC experiences. EOLC stress, calling, and resilience were identified as predictors of EOLC performance and explained 28.3% of its variance.

The results of this study shed light on the need for strategies that help EOLC nurses manage stress, instil a sense of calling, and improve their resilience to improve their EOLC performances. Further, education and intervention programs should be developed to improve EOLC performance, and these programs should be tailored to specific job positions. Additionally, instead of only focusing on providing physical and psychological care, spiritual aspects should be considered in EOLC, and education programs should address spiritual domains. To this end, instruments that can measure spiritual care needs should be developed, and the clinical environment should be altered to enable spiritual care.

## Data Availability

The datasets generated and/or analysed during the current study are not publicly available but are available from the corresponding author on reasonable request.

## Abbreviation

EOLC: End of life care
